# Diagnostic Value of Machine Learning Models in Inflammation of Unknown Origin

**DOI:** 10.3390/jcm14197116

**Published:** 2025-10-09

**Authors:** Selma Özlem Çelikdelen, Onur Inan, Sema Servi, Reyhan Bilici

**Affiliations:** 1 Department of Internal Medicine, Konya City Hospital, University of Health Sciences, 42020 Konya, Türkiye; 2 Faculty of Technology, Department of Computer Engineering, Selçuk University, 42250 Konya, Türkiye; 3 Division of Rheumatology, Department of Internal Medicine, Konya City Hospital, University of Health Sciences, 42020 Konya, Türkiye

**Keywords:** inflammation of unknown origin, machine learning, diagnostic artificial intelligence

## Abstract

**Background:** Inflammation of unknown origin (IUO) represents a persistent clinical challenge, often requiring extensive diagnostic efforts despite nonspecific inflammatory findings such as elevated C-reactive protein (CRP) and erythrocyte sedimentation rate (ESR). The complexity and heterogeneity of its etiologies—including infections, malignancies, and rheumatologic diseases—make timely and accurate diagnosis essential to avoid unnecessary interventions or treatment delays. **Objective**: This study aimed to evaluate the potential of machine learning (ML)-based models in distinguishing the major etiologic subgroups of IUO and to explore their value as clinical decision support tools. **Methods**: We retrospectively analyzed 300 IUO patients hospitalized between January 2023 and December 2024. Four binary one-vs-rest Linear Discriminant Analysis (LDA) models were first developed to independently classify infection, malignancy, rheumatologic disease, and undiagnosed cases using clinical and laboratory parameters. In addition, a multiclass LDA framework was constructed to simultaneously differentiate all four diagnostic groups. Each model was evaluated across 10 independent runs using standard performance metrics, including accuracy, sensitivity, specificity, precision, F1 score, and negative predictive value (NPV). **Results**: The malignancy model achieved the highest performance, with an accuracy of 91.7% and specificity of 0.96. The infection model demonstrated high specificity (0.88) and NPV (0.86), supporting its role in ruling out infection despite lower sensitivity (0.71). The rheumatologic model showed high sensitivity (0.81) but lower specificity (0.73), reflecting the clinical heterogeneity of autoimmune conditions. The undiagnosed model achieved very high accuracy (96.7%) and specificity (0.98) but limited precision and recall (0.50 each). The multiclass LDA framework reached an overall accuracy of 73.3% (mean 66%) with robust specificity (0.90) and NPV (0.89). **Conclusions**: ML-based LDA models demonstrated strong potential to support the diagnostic evaluation of IUO. While malignancy and infection could be predicted with high accuracy, rheumatologic diseases required integration of additional serological and clinical data. These models should be viewed not as stand-alone diagnostic tools but as complementary decision-support systems. Prospective multicenter studies are warranted to externally validate and refine these approaches for broader clinical application.

## 1. Introduction

Inflammation of unknown origin is a challenging clinical entity characterized by persistently elevated acute-phase reactants, such as CRP and/or ESR, in the absence of an immediately identifiable etiology [1]. It encompasses patients who demonstrate biochemical evidence of systemic inflammation without a definitive underlying cause, despite thorough clinical assessment, laboratory investigations, and imaging studies. IUO is encountered more frequently in older adults and individuals with multiple comorbidities, and it is commonly associated with considerable diagnostic uncertainty, therapeutic complexity, and delays in initiating appropriate treatment [2].

The most common etiologies of IUO include malignancies (particularly hematologic neoplasms), infectious diseases (such as tuberculosis and endocarditis), and systemic inflammatory or autoimmune disorders (such as vasculitis and connective tissue diseases) [3]. Differentiating among these three major etiologic categories is critically important not only for determining the patient’s prognosis but also because each group requires a fundamentally different therapeutic approach. For instance, administering immunosuppressive therapy in an infectious IUO case may lead to serious complications, whereas delaying such treatment in malignant or autoimmune conditions could result in inevitable disease progression [1].

The diagnosis of IUO represents one of the most challenging areas in clinical medicine. The diagnostic process often begins with basic laboratory tests; however, in a significant number of cases, advanced and costly investigations such as thoracoabdominal computed tomography, magnetic resonance imaging, endoscopic evaluations, tissue biopsies, and even positron emission tomography (PET-CT) are required [4]. This process leads to significant resource utilization for the healthcare system and imposes both physical and psychological burdens on the patient. Delayed diagnosis may result in adverse outcomes, including disease progression, development of complications, and an increased risk of mortality [5,6,7]. In this context, identifying the underlying cause of IUO through an early, accurate, and systematic approach can positively influence the course of the disease while also enabling more efficient use of healthcare resources. In recent years, there has been growing interest in clinical decision support systems and artificial intelligence (AI)-based approaches to assist diagnostic processes. AI, particularly through machine learning (ML) and deep learning algorithms, possesses strong potential to extract meaningful patterns from large and complex datasets [8,9]. This capability can offer significant contributions in complex diagnostic scenarios such as IUO, where the etiological spectrum is broad and the diagnostic process is highly challenging. Since 2020, the integration of ML algorithms into clinical decision support systems has accelerated, yielding promising results in areas such as diagnosis, classification, and prediction of treatment response [8,9]. In the context of IUO, the use of these advanced analytical approaches may reveal patterns that could be overlooked by conventional methods, thereby enabling faster and more accurate diagnostic possibilities. These advancements may pave the way for an innovative paradigm shift in the clinical management of IUO.

In infectious diseases, particularly in time-sensitive conditions such as sepsis, AI models have shown promising results in early diagnosis and risk stratification. For example, Padoan et al. (2025) [10] investigated a machine learning approach using ESR levels to assess acute infection and demonstrated that sedimentation rates observed in the sepsis/acute inflammatory group were significantly different from those in other groups. These findings suggest that ESR may serve as a potential biomarker [10]. Similarly, the effectiveness of AI-based approaches has also been demonstrated in complex rheumatologic diseases such as rheumatoid arthritis (RA), which require early diagnosis. Momtazmanesh et al. (2022) emphasized the growing potential of artificial intelligence in the screening, diagnosis, and monitoring of RA [11]. In the diagnosis of malignancies, several studies have shown that artificial intelligence models can integrate laboratory parameters, radiologic imaging, and clinical data to support early diagnosis and risk stratification [12].

Today, the growing volume of data and the increasing complexity of clinical decision-making are pushing the limits of traditional diagnostic approaches. This study aims to demonstrate the potential of artificial intelligence-based models in identifying the underlying etiologic causes of IUO, a major diagnostic challenge in internal medicine practice. Using machine learning and deep learning algorithms, the study evaluated the effectiveness of integrated analysis of clinical and laboratory data in differentiating infection, malignancy, and rheumatologic diseases. This approach aims to offer an innovative perspective on the diagnostic process of complex conditions like IUO and to promote more effective use of clinical decision support systems in the future.

## 2. Materials and Methods

### 2.1. Study Design

This retrospective study was conducted by reviewing the electronic medical records of patients admitted to the Internal Medicine and Rheumatology departments of Konya City Hospital between January 2023 and December 2024. During this period, a total of 2083 patient files were screened. Among these, 300 patients aged 18 to 80 years who were hospitalized with a preliminary diagnosis of IUO and met the defined diagnostic criteria were included in the study.

### 2.2. Patient Selection

The diagnosis of IUO was established according to the following criteria:

- Presence of a disease with inflammatory features persisting for at least three weeks;
- Body temperature not exceeding 38.3 °C during at least three separate clinical visits;
- C-reactive protein level above 7 mg/L and/or ESR exceeding the expected value (calculated as age/2 for men and [age + 10]/2 for women) in at least three visits;
- Failure to reach a definitive diagnosis despite at least three days of hospitalization or three outpatient evaluations with detailed investigations [1,13].

**Figure 1:**
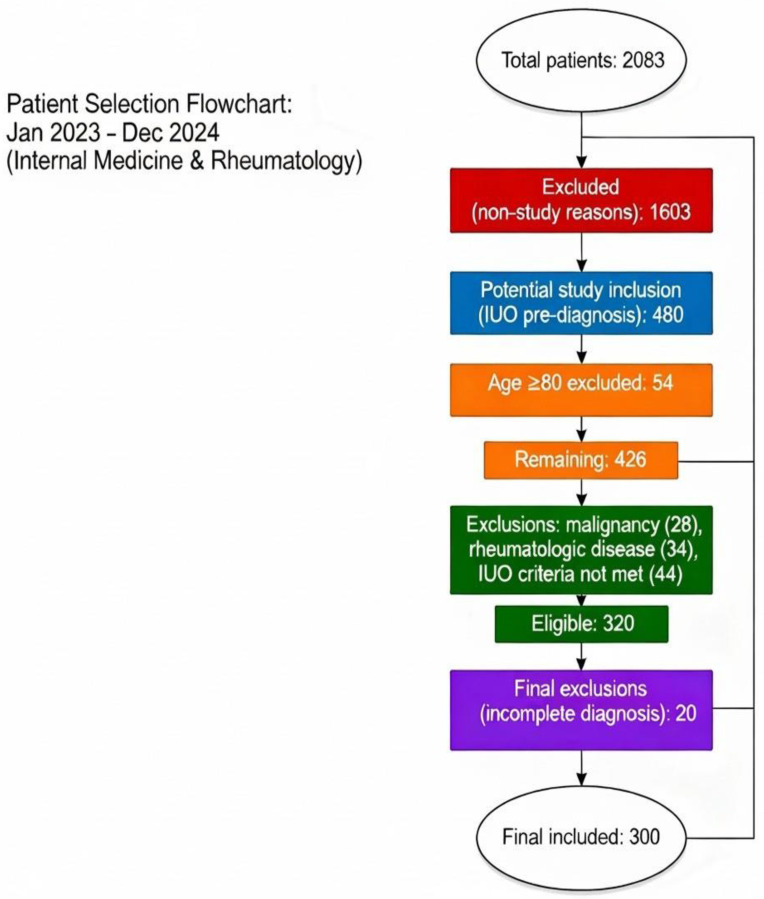
Patient Selection Flowchart.

Of the total 2083 hospitalized patients during the study period, 1603 were excluded due to non-IUO causes of admission. Patients older than 80 years (*n* = 54) were excluded due to the higher prevalence of comorbidities, frailty, and atypical clinical presentations that could increase heterogeneity in this population. Further exclusions were applied to patients with a prior history of rheumatologic disease (*n* = 34), known malignancy (*n* = 28), or failure to fulfill the predefined IUO diagnostic criteria (*n* = 44). In addition, 20 patients were excluded because they were discharged before completing the diagnostic workup or declined further investigations, precluding confirmation of the diagnosis (Figure 1).

Demographic information (age, sex), presenting complaints, laboratory findings (ESR, CRP, complete blood count parameters, creatinine, albumin, alanine aminotransferase [ALT], aspartate aminotransferase [AST], lactate dehydrogenase [LDH], uric acid), radiologic imaging results, and, if available, biopsy and pathology reports were retrospectively collected from the hospital information management system. In addition, discharge summaries were reviewed to determine and document the final diagnoses of the patients.

This study was approved by the Ethics Committee of KTO Karatay University Faculty of Medicine on 28 November 2024, with approval number 2024/019.

### 2.3. Statistical Analysis

The statistical analyses of the study were conducted in two stages:

1. Conventional Statistical Analyses (using SPSS)

Descriptive analyses were performed using IBM SPSS Statistics version 27.0 (IBM Corp., Armonk, NY, USA). Continuous variables were expressed as mean ± standard deviation or median (interquartile range), while categorical variables were presented as counts and percentages (%). Descriptive statistics were calculated for demographic data, presenting complaints, and laboratory parameters.

2. Machine Learning-Based Analyses (LDA Models)

In this study, four separate LDA models were developed to differentiate among infection, malignancy, rheumatologic disease, and undiagnosed conditions in patients with IUO. LDA is a supervised learning algorithm that performs classification by generating linear combinations of features to maximize separation between predefined classes [14].

In the first phase, a one-vs-rest strategy was applied to evaluate each of the four diagnostic categories. Accordingly, four independent LDA models were constructed and implemented as binary classifiers: the infection model classified patients as “infection” versus “non-infection”, the malignancy model as “malignancy” versus “non-malignancy”, the rheumatologic model as “rheumatologic” versus “non-rheumatologic”, and the undiagnosed model as “undiagnosed” versus “diagnosed”. Each model incorporated a broad set of predictors, including complete blood count parameters (WBC, lymphocytes, neutrophils, platelets, hemoglobin), biochemical markers (LDH, albumin, creatinine, uric acid, ALT, AST, CRP, ESR), clinical symptoms (fever, weight loss), sex and age (Supplementary Material Section S2). Model outputs indicated the probability of presence (coded as 1) or absence (coded as 0) of the respective diagnostic class. All LDA model constructions and computations were performed using MATLAB R2015b.

In the second phase of the study, a multiclass LDA framework was developed to simultaneously classify all four diagnostic categories—infection, malignancy, rheumatologic disease, and undiagnosed cases—within a single model. Using all available clinical and biochemical variables, the model computed separate linear discriminant functions and coefficients for each class (Supplementary Material Section S2.5). For each patient, the feature values were entered into these functions, and the class with the highest discriminant score was selected as the predicted outcome. Model outputs were expressed as numerical labels (1–4), corresponding to infection (1), malignancy (2), rheumatologic disease (3) or undiagnosed condition (4).

For model training and testing, the dataset was randomly split, and each model was executed 10 times using different random seeds. In each run, the model was retrained and tested independently. Performance was assessed using standard classification metrics, including accuracy, precision, recall, F1 score, specificity, negative predictive value (NPV) and positive predictive value (PPV). To evaluate stability, the results from the 10 independent runs were aggregated, and the mean, maximum, minimum and standard deviation values of each performance metric were reported.

## 3. Results

### 3.1. Patient Characteristics and Diagnostic Groups

In this study, 300 patients with IUO were evaluated. Of these, 51% were male (*n* = 153) and 49% were female (*n* = 147). The mean age was calculated as 59.42 ± 16.16 years. Among the laboratory parameters, the median ESR was 70 mm/hour (IQR: 41.75), and the median CRP level was 76.85 mg/L (IQR: 95.30). The general characteristics of the patients’ clinical and laboratory parameters are presented in Table 1.

When evaluating the symptoms at hospital admission, elevated acute-phase reactants (APRs) were the most common reason for hospitalization, observed in 72.3% of the patients. Other common symptoms included weight loss (15%) and elevated body temperature (10.6%). The most common diagnostic group was rheumatologic diseases (*n* = 122, 40.7%), followed by malignancies (*n* = 74, 24.7%) and infections (*n* = 69, 23%). In 35 patients (11.7%), no definitive diagnosis could be established despite advanced diagnostic investigations. The distribution of patients based on reasons for hospitalization and their final diagnoses is presented in Table 2.

### 3.2. Results of Machine Learning Analysis

A total of four independent LDA models were developed and evaluated to distinguish infection, malignancy, rheumatologic disease, and undiagnosed conditions in patients with IUO. In the first phase, the one-vs-rest strategy enabled binary classification for each diagnostic category, with model outputs representing the probability of presence or absence of the respective condition. In the second phase, a multiclass LDA framework was implemented to simultaneously classify all four diagnostic groups within a single analysis. This design allowed for both focused evaluation of individual disease categories and a comprehensive assessment of the model’s overall discriminative performance across multiple etiologies.

In our study, the LDA model for predicting infection demonstrated an overall accuracy of 86.67% and a specificity of 0.88, while sensitivity (0.71) and precision (0.45) were comparatively lower. When evaluated across 10 independent runs, the model achieved a mean accuracy of 76.83% (Supplementary Table S1). Importantly, the consistently high specificity (0.84) and NPV (0.86) highlight the model’s strength in reliably ruling out infection. Although its capacity to correctly identify infected patients was limited, the model proved effective as a decision-support tool for excluding infection in the differential diagnostic process.

The LDA model developed to predict the presence of malignancy achieved the highest accuracy (91.67%) and specificity (0.96) among the four LDA models. In addition, other performance metrics such as precision (0.78) and NPV (0.94) were also found to be high. The model achieved an average accuracy of 85.83% across 10 independent runs (Supplementary Table S2). These results highlight the model as a balanced and reliable classifier in distinguishing malignancy.

The LDA model developed for predicting rheumatologic diseases demonstrated lower accuracy (76.67%) and specificity (0.7368) compared to the infection and malignancy classification models. The model achieved an average accuracy of 69.00% across 10 independent runs (Supplementary Table S3). The high sensitivity (0.81) and NPV (0.87) indicate that the model was effective in identifying individuals with rheumatologic disease, but demonstrated moderate performance in excluding individuals without the condition.

Of the 300 patients included in the study, 35 were classified into the undiagnosed group. Despite the relatively small number of undiagnosed cases, the LDA model developed for this group achieved high accuracy (96.67%) and specificity (0.98). However, both sensitivity (0.50) and precision (0.50) were found to be low. The model achieved an average accuracy of 89.67% across 10 independent runs (Supplementary Table S4). The model was found to be effective in accurately excluding the other three disease groups (infection, malignancy, and rheumatologic disease) within the undiagnosed cohort.

**Figure 2:**
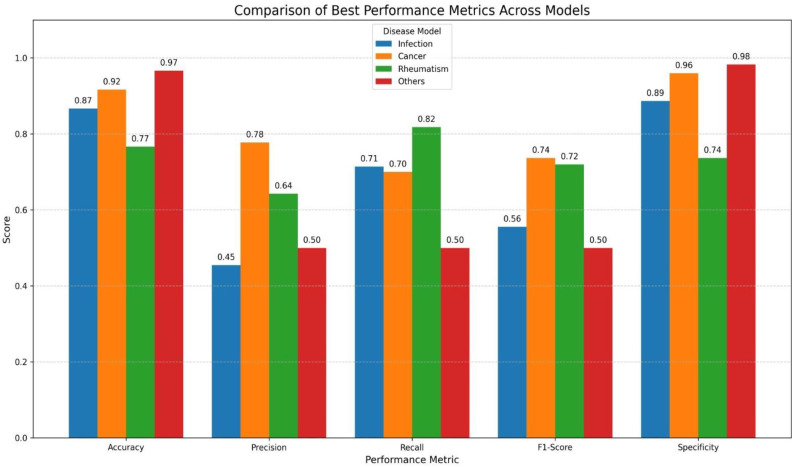
Graphical Visualization of Best Performance Results of LDA Models Across Diagnostic Subgroups. *X*-axis shows classification metrics (accuracy, precision, recall, F1 score and specificity) and *Y*-axis represents performance scores (range 0–1). Colored bars display results for each disease model (infection, malignancy/cancer, rheumatologic disease and undiagnosed/others).

The average and best performance metrics of the LDA models for all diagnostic subgroups are summarized in Table 3, Figure 2 and Table 4, respectively.

**Figure 3:**
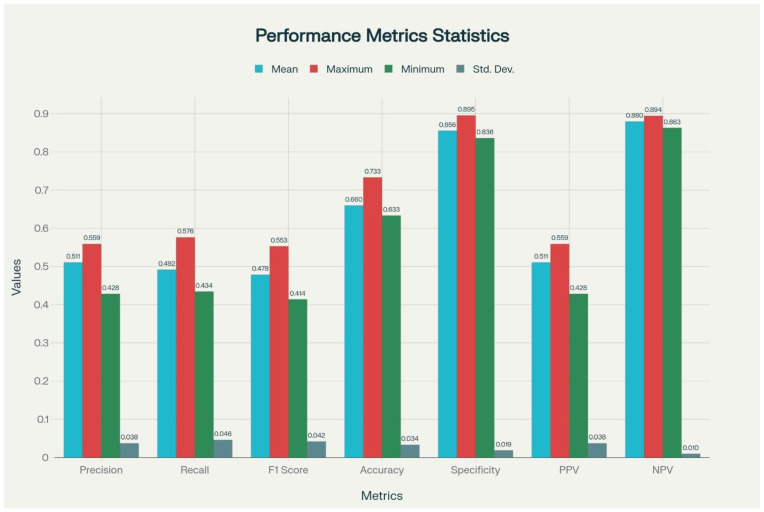
Graphical Visualization of Performance Metrics of the Multiclass LDA Model Across 10 Runs. PPV: Positive Predictive Value; NPV: Negative Predictive Value. F1-Score: Harmonic mean of precision and recall. Calculated as: 2 × [(precision × recall)/precision + recall)]. *X*-axis shows performance metrics (precision, recall, F1 score, accuracy, specificity, PPV and NPV) and *Y*-axis represents performance scores (range 0–1). Colored bars display mean, maximum, minimum and standard deviation for each metric.

In the final phase of the study, beyond the one-vs-rest evaluations, a multiclass LDA model was developed to simultaneously classify infection, malignancy, rheumatologic disease, and undiagnosed conditions. This approach enabled assessment of the model’s ability to distinguish across all four diagnostic groups within a single analysis, thereby providing a more comprehensive evaluation of overall performance. The multiclass LDA model achieved an overall accuracy of 73.33% in its best-performing run performance metrics included a precision of 0.56, recall of 0.58, F1 score of 0.55, specificity of 0.90, PPV of 0.56 and NPV of 0.89. To evaluate model stability, 10 independent runs were conducted, yielding an average accuracy of 66% (Table 5, Figure 3). In particular, the low standard deviations observed for accuracy, specificity, and NPV highlighted the robustness and reproducibility of the model.

## 4. Discussion

Inflammation of unknown origin remains one of the most complex and challenging diagnostic areas in clinical practice. Despite advances in imaging and laboratory technologies, diagnostic delays are common in IUO cases and often lead to the use of costly diagnostic algorithms [15]. There is a growing need for more innovative strategies, alternative clinical perspectives, and systematic evaluation algorithms in the differential diagnosis of IUO. In this study, clinical and laboratory data from 300 patients evaluated for IUO were used to develop machine learning-supported LDA models aimed at differentiating the most common etiologic groups, including infection, malignancy and rheumatologic diseases, and to assess the performance of these models.

In this study, we applied both binary and multiclass LDA frameworks to differentiate the major etiologic subgroups of IUO. While the one-vs-rest strategy allowed focused evaluation of individual categories, the multiclass approach enabled simultaneous classification across all four groups, providing a more comprehensive assessment of overall diagnostic performance.

The model developed to predict the presence of infection demonstrated high specificity and negative predictive value, while showing lower performance in terms of precision and sensitivity. This indicates that the model was effective in excluding non-infected individuals but was somewhat limited in identifying those who were actually infected. In a study conducted by Padoan et al. (2025) [10], ML models based on ESR levels were used with the aim of distinguishing individuals with sepsis and acute inflammatory conditions. The model developed in this context achieved notably high performance in terms of accuracy and specificity. However, the patient population targeted by this model represents only a narrow subset of the infection spectrum, specifically those with severe and systemic presentations. In contrast, the model developed in our study aims to predict the presence of infection within a broader and more heterogeneous IUO population. Therefore, while our model demonstrated high specificity and negative predictive value in excluding non-infected individuals across the general infection spectrum, it showed relatively limited sensitivity in identifying infected cases. These findings highlight the variability that may arise across different datasets and methodological approaches, while also suggesting that models with high negative predictive power may offer valuable contributions in clinical scenarios characterized by diagnostic uncertainty, such as IUO [10]. Indeed, during the etiologic evaluation of IUO patients, empirical antibiotic therapy is often initiated before a definitive diagnosis is established [16]. In this context, the ability of an ML-based model to rule out infection may help reduce unnecessary antibiotic use, thereby contributing to efforts against antibiotic resistance and enhancing patient safety. In a systematic review and meta-analysis published by Pennisi et al. in 2025, it was emphasized that artificial intelligence models operate with high predictive accuracy in antimicrobial management, thereby enabling the development of more targeted treatment strategies [17].

Another important finding of our study was the high performance of the model in distinguishing malignancy cases. The developed model demonstrated the highest performance among the four LDA models, with high accuracy, specificity, precision, and NPV. This finding may be attributed to the fact that malignancies often exhibit more distinct inflammatory biomarker patterns. Indeed, a study conducted on patients with colorectal cancer reported that ML models based on inflammatory biomarkers could be used with high accuracy to predict postoperative survival [18]. Additionally, several studies have demonstrated that ML techniques can generate highly specific models for applications such as risk stratification and prognosis prediction in hematologic malignancies [19]. In a review published in 2024, growing evidence was presented that artificial intelligence algorithms can significantly improve diagnostic accuracy, early detection rates, and overall patient management in the diagnosis of malignancies [20].

In our study, the lowest performance was observed in the model developed for predicting rheumatologic diseases. This model demonstrated high sensitivity and negative predictive value; however, its accuracy and specificity were relatively low. This suggests that the model was effective in identifying individuals with rheumatologic disease but showed limited success in excluding those without the condition. The model’s ability to detect rheumatologic disease was also found to be lower than that of the infection and malignancy models. This may be attributed to the clinical heterogeneity of autoinflammatory and autoimmune diseases, variability in laboratory findings, and the critical role of clinical history and physical examination findings in the diagnostic process. Despite substantial advances in diagnostic technology, accurate identification of rheumatologic conditions still relies primarily on meticulous clinical history and thorough physical examination, while algorithm-based approaches should be regarded as complementary rather than standalone diagnostic methods [21]. Similarly, studies evaluating the use of ML-based models in autoimmune diseases have shown that existing datasets often fail to fully capture clinical heterogeneity, and that physical examination findings and autoantibody panel data are frequently incomplete or lacking [22,23]. These findings indicate that, for AI models to be successful in the differential diagnosis of rheumatologic diseases, it is essential to integrate not only laboratory data but also clinical symptoms, physical examination findings, and autoantibody testing into a multidimensional dataset. There is strong evidence in the literature that the combined evaluation of multimodal data, including laboratory results, clinical symptoms, and imaging findings, can significantly improve model performance [24].

The model developed for the undiagnosed IUO group showed high accuracy and specificity; however, its precision and recall rates were found to be low. This suggests that the model was successful in excluding the three major etiologic groups within this cohort, but had limited ability to directly predict undiagnosed cases. These findings may be related to the clinical heterogeneity of the undiagnosed group and the possible presence of cases that received incomplete or missed diagnoses.

In our study, the multiclass LDA model enabled the simultaneous classification of four etiological groups within a single analysis. It achieved an overall accuracy of 73.3% in its best-performing run, and the consistently low standard deviations in specificity and negative predictive value supported its robustness across different data subsets. These findings suggest that the multiclass approach may be useful as a preliminary screening tool in complex clinical scenarios where multiple potential diagnoses must be considered. In contrast, the binary one-vs-rest models demonstrated superior performance, particularly the malignancy (91.7%) and infection (86.7%) models, which captured disease-specific biomarker patterns more effectively. This highlights the greater diagnostic precision and stability of specialized binary models, which may be prioritized in clinical decision support systems.

Building on these observations, our study further demonstrated the potential of ML-based LDA models in the differential diagnosis of IUO cases. Specifically, malignancy and infection could be classified with high accuracy using laboratory and clinical data, whereas more heterogeneous conditions such as rheumatologic diseases appeared to require integration of additional physical examination findings and specific serological markers. For undiagnosed cases, the model effectively excluded other etiologies but showed limited capacity for direct prediction.

Overall, these results indicate that artificial intelligence-based approaches may serve as valuable guidance tools for clinicians in the diagnostic evaluation of IUO, while underscoring the indispensable role of clinical judgment. Accordingly, such models should be regarded not as stand-alone diagnostic instruments, but rather as complementary decision-support systems with potential future utility in clinical practice.

The strengths of this study include the systematic evaluation of a highly heterogeneous patient population investigated for IUO, the combined analysis of a broad range of clinical and biochemical variables, and the validation of the developed models through 10 independent runs to ensure reliability. Beyond the development of binary one-vs-rest classifiers, another major strength lies in the integration of a multiclass LDA framework capable of simultaneously distinguishing all four diagnostic categories—infectious, malignant, rheumatologic and undiagnosed conditions—within a single analysis. This multiclass approach provided a more holistic assessment of model performance and reflected the inherently complex and multidimensional nature of IUO more realistically. By enabling concurrent evaluation of multiple potential etiologies, the multiclass model highlights an innovative methodological contribution that extends beyond disease-specific classification and demonstrates the potential utility of AI-based systems as comprehensive decision-support tools in clinical practice.

However, the study also has several limitations. First, its single-center and retrospective design may restrict the generalizability of the findings, as the patient population and clinical practices may not fully represent broader and more diverse healthcare settings. Additionally, the retrospective nature of the data limited our ability to incorporate detailed physical examination findings, which are particularly critical in patient groups such as those with rheumatologic diseases. The relatively small number of cases in the undiagnosed IUO subgroup may have reduced the learning capacity of the corresponding model, leading it to perform better in excluding other etiologies rather than directly identifying undiagnosed cases.

Another important limitation is the absence of external validation. While the models were internally validated through 10 independent runs, external validation in independent cohorts and diverse populations is essential to establish generalizability, strengthen clinical reliability, and increase physicians’ confidence in adopting such models. As this study represents one of the first attempts to develop and internally validate ML-based LDA models for the classification of IUO, these results should be interpreted as preliminary. Future multicenter, prospective studies with larger and more heterogeneous patient groups will be crucial to confirm the robustness of the models, refine their predictive capacity, and enhance their clinical applicability.

Despite these limitations, our study provides an important foundation for diagnostic modeling in IUO and demonstrates the potential of artificial intelligence-based decision support systems. These findings may serve as a valuable starting point for the development of data-driven approaches in the differential diagnosis of infection, malignancy, and rheumatologic diseases, paving the way for more reliable and clinically applicable tools in future practice.

## 5. Conclusions

In conclusion, this study demonstrates that ML-based LDA models can provide valuable support to clinicians in the diagnostic management of IUO. Our findings highlight that while malignancy and infection can be predicted with high accuracy using laboratory and clinical parameters, more heterogeneous groups such as rheumatologic diseases require the integration of additional clinical and serological data. As one of the first attempts at AI-based diagnostic modeling in IUO, this study underscores that such models should be regarded not as stand-alone diagnostic tools, but as complementary systems to guide clinical decision-making. Future multicenter, prospective studies with larger and more comprehensive datasets will be essential to enhance generalizability, strengthen reliability, and ultimately facilitate the broader adoption of AI-supported approaches in the management of IUO.

## Figures and Tables

**Table 1:** General Characteristics and Laboratory Parameters of Patients Evaluated for Inflammation of Unknown Origin (IUO).

Parameter	Values or Count/Percentages
Demographic Characteristics	
Age, years	59.42 ± 16.16
Female, *n* (%)	147 (49.0%)
Laboratory Parameters	
ESR, mm/h	70 (IQR: 41.75)
CRP, mg/L	76.85 (IQR: 95.30)
Creatinine, mg/dL	1.00 ± 0.76
Albumin, g/L	3.41 ± 0.64
Uric acid, mg/dL	5 (IQR: 2.6)
ALT, U/L	16 (IQR: 18)
AST, U/L	20 (IQR: 18)
LDH, U/L	237 (IQR: 129)
Hemoglobin, g/dL	10.93 ± 2.35
Platelets, ×10^3^/μL	301 (IQR: 174.25)
White blood cells, ×10^3^/μL	9.22 (IQR: 5.11)
Neutrophils, ×10^3^/μL	6.12 (IQR: 4.6)
Lymphocytes, ×10^3^/μL	1.70 (IQR: 1.13)

Continuous variables are expressed as mean ± standard deviation (SD) or median with interquartile range (IQR) depending on distribution. Categorical variables are presented as count and percentage. ESR: Erythrocyte Sedimentation Rate; CRP: C-reactive protein; ALT: Alanine aminotransferase; AST: Aspartate aminotransferase; LDH: Lactate dehydrogenase.

**Table 2:** Presenting Symptoms and Diagnostic Outcomes in Patients Evaluated for IUO.

Final Diagnosis	Number of Patients (*n* = 300)	Frequency (%)
Infection	69	23.0
Malignancy	74	24.7
Rheumatologic disease	122	40.7
Undiagnosed	35	11.7
**Primary presenting complaint**		
APR *	217	72.3
Weight loss	45	15.0
Fever (<38.3 °C)	32	10.6
Other symptoms	20	6.6

APR: Elevated acute-phase reactants. Among 300 patients, 205 presented solely with elevated APR, 37 with only weight loss, and 25 with only fever. Additionally, 6 patients had both elevated APR and weight loss, 5 had elevated APR and fever, 1 had both fever and weight loss, and 1 had all three symptoms simultaneously. A total of 20 patients were admitted with constitutional symptoms. * Elevated acute-phase reactants (CRP and/or ESR) were present in all patients as part of the inclusion criteria. The numbers and percentages shown in the table indicate the patients for whom abnormal APR levels constituted the primary presenting complaint at admission.

**Table 3:** Best Performance Results of LDA Models for Each Diagnostic Subgroup.

Group	Accuracy (%)	Precision	Recall	F1 Score	Specificity	NPV	PPV
Infection	86.67	0.45	0.71	0.55	0.88	0.95	0.45
Malignancy	91.67	0.78	0.70	0.73	0.96	0.94	0.77
Rheumatologic	76.67	0.64	0.81	0.72	0.73	0.87	0.64
Undiagnosed	96.67	0.50	0.50	0.50	0.98	0.98	0.50

PPV: Positive Predictive Value; NPV: Negative Predictive Value. F1-Score: Harmonic mean of precision and recall. Calculated as: 2 × [(precision × recall)/precision + recall)]. Note: This table presents the best-performing results for each LDA model. All models achieved high specificity and NPV, demonstrating strong ability to distinguish non-target (negative) classes. The malignancy model had the highest accuracy (91.67%) and overall balance, while the infection model showed the lowest variability across runs. The undiagnosed model, despite class imbalance, achieved 96.67% accuracy, emphasizing its strength in correctly classifying negative cases.

**Table 4:** Average Performance of LDA Models Across Diagnostic Subgroups.

Metric	Infection	Malignancy	Rheumatologic	Undiagnosed
Accuracy (%)	76.83	85.83	69.00	89.67
Precision	0.47	0.79	0.63	0.35
Recall	0.47	0.60	0.75	0.34
F1-Score	0.45	0.68	0.68	0.34
Specificity	0.84	0.93	0.63	0.94
PPV	0.47	0.79	0.63	0.35
NPV	0.86	0.87	0.76	0.94

PPV: Positive Predictive Value; NPV: Negative Predictive Value; F1-Score: Harmonic mean of precision and recall. Calculated as: 2 × [(precision × recall)/precision + recall)]. Note: This table summarizes the average performance metrics of LDA models used to predict diagnostic subgroups in IUO patients. Each column represents a different classification model trained to identify one specific diagnostic group (positive class = 1).

**Table 5:** Performance Metrics of the Multiclass LDA Model Based on 10 Independent Runs.

Metric	Mean	Range (Min–Max)	Standard Deviation
Accuracy (%)	66.00	63.33–73.33	0.03
Precision	0.51	0.42–0.55	0.03
Recall (Sensitivity)	0.49	0.43–0.57	0.04
F1-Score	0.47	0.41–0.55	0.04
Specificity	0.85	0.83–0.89	0.01
PPV	0.51	0.42–0.55	0.03
NPV	0.87	0.86–0.89	0.01

PPV: Positive Predictive Value; NPV: Negative Predictive Value. F1-Score: Harmonic mean of precision and recall. Calculated as: 2 × [(precision × recall)/precision + recall)]. Note: The multiclass LDA model, designed to classify IUO patients into four etiologic groups, achieved a mean accuracy of 66.0% across 10 independent runs. While specificity (0.85) and NPV (0.87) were relatively high, indicating strength in ruling out incorrect classes, precision, recall, and F1-scores were modest, reflecting the challenge of multiclass prediction. Low standard deviations across metrics support the stability of the model despite moderate overall accuracy.

## Data Availability

The datasets generated during and/or analyzed during the current study are available from the corresponding author on reasonable request.

## Supplementary Materials

The following supporting information can be downloaded at: https://www.mdpi.com/article/10.3390/jcm14197116/s1, Supplementary Material Section S1. Performance Metrics of LDA-Based Models for Predicting Diagnostic Categories in Patients with Inflammation of Unknown Origin (IUO). This supplementary table presents detailed performance metrics (mean, range, standard deviation) of four LDA models used to classify IUO cases as infection, malignancy, rheumatologic disease, or undiagnosed. Metrics include accuracy, precision, recall, F1-score, specificity, PPV, and NPV across 10 independent runs. Supplementary Table S1. Performance Metrics of the LDA Model for Infection Prediction Based on 10 Independent Runs. Supplementary Table S2. Performance Metrics of the LDA Model for Malignancy Prediction Based on 10 Independent Runs. Supplementary Table S3. Performance Metrics of the LDA Model for Rheumatologic Disease Prediction Based on 10 Independent Runs. Supplementary Table S4. Performance Metrics of the LDA Model for Undiagnosed IUO Prediction Based on 10 Independent Runs. Supplementary Material Section S2. Discriminant Function Equations of LDA Models for Predicting Diagnostic Categories in IUO. This file provides the mathematical discriminant function equations (Y) derived from LDA models used for classifying IUO patients into four diagnostic categories. Each equation represents the weighted contribution of clinical and laboratory variables for each class. S2.1. Infection Prediction Model. S2.2. Malignancy Prediction Model. S2.3. Rheumatologic Disease Prediction Model. S2.4. Undiagnosed IUO Prediction Model. S2.5. Multiclass LDA Model.

## Abbreviations

APRs	Acute-phase reactants
AI	Artificial intelligence
ALT	Alanine aminotransferase
AST	Aspartate aminotransferase
CRP	C-reactive protein
ESR	Erythrocyte sedimentation rate
IUO	Inflammation of unknown origin
LDA	Linear discriminant analysis
LDH	Lactate dehydrogenase
ML	Machine learning
NPV	Negative predictive value
PET-CT	Positron emission tomography–computed tomography
PPV	Positive predictive value
RA	Rheumatoid arthritis
