# Supplementary material for: Diagnostic Value of Machine Learning Models in Inflammation of Unknown Origin
Source: J Clin Med. 2025 Oct 9;14(19):7116. doi: 10.3390/jcm14197116 (PMC12526033; doi:10.3390/jcm14197116)
Supplement: Supplementary file 1 [file jcm-14-07116-s001.zip › jcm-3881611-supplementary.pdf]

**Supplementary Material  Section S1.**

**Performance Metrics of LDA-Based Models for Predicting Diagnostic Categories in Patients with Inflammation of Unknown Origin (IUO)**

This supplementary table presents the performance metrics of four separate Linear Discriminant Analysis (LDA) models developed to predict infection, rheumatologic disease, malignancy, and undiagnosed status in patients with IUO. Each model was trained and evaluated over 10 independent runs using clinical and laboratory parameters. Reported metrics include mean accuracy, precision, recall (sensitivity), F1-score, specificity, positive predictive value (PPV), and negative predictive value (NPV), along with their respective ranges and standard deviations.

These results support the potential utility of LDA-based machine learning models as decision support tools in differentiating diagnostic categories among IUO cases.

**Supplementary Table S1. Performance Metrics of the LDA Model for Infection Prediction Based on 10 Independent Runs**

| **Metric** | **Mean** | **Range (Min–Max)** | **Standard Deviation** |
|---|---|---|---|
| Accuracy (%) | 76.83 | 65.00 – 86.67 | 7.13 |
| Precision | 0.47 | 0.28 – 0.85 | 0.20 |
| Recall (Sensitivity) | 0.47 | 0.40 – 0.71 | 0.09 |
| F1-Score | 0.45 | 0.34 – 0.55 | 0.08 |
| Specificity | 0.84 | 0.68 – 0.97 | 0.09 |
| PPV | 0.47 | 0.28 – 0.85 | 0.20 |
| NPV | 0.86 | 0.79 – 0.95 | 0.04 |

PPV: Positive Predictive Value; NPV: Negative Predictive Value. F1-Score: Harmonic mean of precision and recall. Calculated as: 2x [ (precision x recall)/ precision+recall) ]

**Note:** The LDA model was developed to predict infection (1) or non-infection (0) in IUO patients using clinical and laboratory features. In 10 independent runs, it achieved a mean accuracy of 76.83% and a maximum of 86.67%. High specificity (0.84) and NPV (0.86) indicate strong performance in ruling out infection. Low variability across metrics supports the model’s stability.

**Supplementary Table S2. Performance Metrics of the LDA Model for Malignancy Prediction Based on 10 Independent Runs**

| **Metric** | **Mean** | **Range (Min–Max)** | **Standard Deviation** |
|---|---|---|---|
| Accuracy (%) | 85.83 | 80.00 – 91.67 | 4.10 |
| Precision | 0.79 | 0.58 – 0.88 | 0.10 |
| Recall (Sensitivity) | 0.60 | 0.50 – 0.75 | 0.07 |
| F1-Score | 0.68 | 0.62 – 0.75 | 0.04 |
| Specificity | 0.93 | 0.84 – 0.97 | 0.04 |
| PPV | 0.79 | 0.58 – 0.88 | 0.10 |
| NPV | 0.87 | 0.80 – 0.94 | 0.03 |

PPV: Positive Predictive Value; NPV: Negative Predictive Value. F1-Score: Harmonic mean of precision and recall. Calculated as: 2x [ (precision x recall)/ precision+recall) ]

**Note:** The LDA model was developed to predict malignancy (1) or non-malignancy (0) in IUO patients using clinical and laboratory features. In 10 independent runs, it achieved a mean accuracy of 85.83% and a maximum of 91.67%. High specificity (0.93) and NPV (0.87) indicate strong performance in ruling out malignancy. Low variability across metrics supports the model's stability.

**Supplementary Table S3. Performance Metrics of the LDA Model for Rheumatologic Disease Prediction Based on 10 Independent Runs**

| Metric | Mean | Range (Min–Max) | Standard Deviation |
|---|---|---|---|
| Accuracy (%) | 69.00 | 61.67 – 76.67 | 5.56 |
| Precision | 0.63 | 0.54 – 0.71 | 0.05 |
| Recall (Sensitivity) | 0.75 | 0.63 – 0.92 | 0.07 |
| F1-Score | 0.68 | 0.65 – 0.72 | 0.02 |
| Specificity | 0.63 | 0.45 – 0.84 | 0.12 |
| PPV | 0.63 | 0.54 – 0.71 | 0.05 |
| NPV | 0.76 | 0.62 – 0.88 | 0.08 |

PPV: Positive Predictive Value; NPV: Negative Predictive Value. F1-Score: Harmonic mean of precision and recall. Calculated as: 2x [ (precision x recall)/ precision+recall) ]

**Note:** The LDA model was developed to predict rheumatologic disease (1) or non-rheumatologic causes (0) in IUO patients using clinical and laboratory features. In 10 independent runs, it achieved a mean accuracy of 69.00% and a maximum of 76.67%. Low variability across metrics supports the model's stability.

**Supplementary Table S4. Performance Metrics of the LDA Model for Undiagnosed IUO Prediction Based on 10 Independent Runs**

| Metric | Mean | Range (Min–Max) | Standard Deviation |
|---|---|---|---|
| Accuracy (%) | 89.67 | 81.67 – 96.67 | 6.61 |
| Precision | 0.35 | 0.25 – 0.50 | 0.12 |
| Recall (Sensitivity) | 0.34 | 0.20 – 0.50 | 0.13 |
| F1-Score | 0.34 | 0.22 – 0.50 | 0.13 |
| Specificity | 0.94 | 0.88– 0.98 | 0.04 |
| PPV | 0.35 | 0.25– 0.50 | 0.12 |
| NPV | 0.94 | 0.90– 0.98 | 0.03 |

PPV: Positive Predictive Value; NPV: Negative Predictive Value. F1-Score: Harmonic mean of precision and recall. Calculated as: 2x [ (precision x recall)/ precision+recall) ]

**Note:** The LDA model was developed to predict undiagnosed IUO cases (1 = undiagnosed, 0 = diagnosed) based on clinical and laboratory variables. Despite class imbalance, the model achieved a maximum accuracy of 96.67%, with consistently high specificity (0.94) and NPV (0.94) across 10 independent runs.

## Supplementary Material Section S2.

### Discriminant Function Equations of LDA Models for Predicting Diagnostic Categories in IUO

This section presents the linear discriminant function (Y) equations obtained from LDA models developed to classify IUO patients into four diagnostic categories: infection, malignancy, rheumatologic disease, and undiagnosed cases. Each equation represents a weighted linear combination of clinical and laboratory variables. A higher Y score increases the likelihood of belonging to the respective class. These models form the basis of the decision boundaries used in classification.

### S2.1.Infection Prediction Model

$$Y=1.5353\cdot\text{LDH}+0.5282\cdot\text{Lymphocyte}+6.7708\cdot\text{Neutrophil}+1.6352\cdot\text{Platelet}-5.4577\cdot\text{WBC}+0.3325\cdot\text{Albumin}-0.1068\cdot\text{Fever}+0.2734\cdot\text{Sex}-3.1140\cdot\text{CRP}+3.0117\cdot\text{ESR}+0.2059\cdot\text{Hemoglobin}+0.6845\cdot\text{Weight loss}-0.2182\cdot\text{Uric acid}+1.1114\cdot\text{Age}-1.3065$$

### S2.2.Malignancy Prediction Model

$$Y=-6.9708\cdot\text{LDH}-2.0513\cdot\text{Lymphocyte}-0.4469\cdot\text{Neutrophil}+0.3857\cdot\text{Platelet}-0.5118\cdot\text{WBC}-2.1079\cdot\text{Albumin}+0.6049\cdot\text{Fever}-1.0088\cdot\text{Sex}+0.0309\cdot\text{CRP}+1.4429\cdot\text{ESR}+4.6401\cdot\text{Hemoglobin}-0.7885\cdot\text{Weight loss}+0.3634\cdot\text{Uric acid}-2.1580\cdot\text{Age}+2.7567$$

### S2.3.Rheumatologic Disease Prediction Model

$$Y=3.1707\cdot\text{LDH}-0.4220\cdot\text{Lymphocyte}-5.0400\cdot\text{Neutrophil}-1.9534\cdot\text{Platelet}+4.6454\cdot\text{WBC}+1.0989\cdot\text{Albumin}-0.3528\cdot\text{Fever}+0.1456\cdot\text{Sex}+2.0892\cdot\text{CRP}-2.5829\cdot\text{ESR}-2.9107\cdot\text{Hemoglobin}-0.1251\cdot\text{Weight loss}-0.4604\cdot\text{Uric acid}+1.3989\cdot\text{Age}+1.6208$$

### S2.4.Undiagnosed IUO Prediction Model

$$Y=4.1025\cdot\text{LDH}+1.9150\cdot\text{Lymphocyte}+1.6189\cdot\text{Neutrophil}+0.9792\cdot\text{Platelet}-1.6911\cdot\text{WBC}-1.4520\cdot\text{Albumin}-0.2433\cdot\text{Fever}+0.5744\cdot\text{Sex}+0.1321\cdot\text{CRP}+0.4876\cdot\text{ESR}+2.0427\cdot\text{Hemoglobin}+0.0524\cdot\text{Weight loss}-0.2066\cdot\text{Uric acid}-0.4515\cdot\text{Age}-0.5381$$

### S2.5. Multiclass LDA Model

$$Y=-6.1859\cdot\text{LDH}-2.0916\cdot\text{Lymphocyte}-5.7363\cdot\text{Neutrophil}-1.4023\cdot\text{Platelet}+3.8124\cdot\text{WBC}-1.2807\cdot\text{Albumin}+0.7308\cdot\text{Fever}-0.5758\cdot\text{Sex}+2.6587\cdot\text{CRP}-1.8002\cdot\text{ESR}+2.6911\cdot\text{Hemoglobin}-1.1118\cdot\text{Weight loss}+0.0095\cdot\text{Uric acid}-2.7458\cdot\text{Age}+3.4002$$
